# P-541. PD-1 expression impacts cardiovascular risk in naïve PLWH (people living with HIV), undergo suppressive ARV

**DOI:** 10.1093/ofid/ofae631.740

**Published:** 2025-01-29

**Authors:** Bogusz J Aksak-Wąs, Miłosz Parczewski, Karolina Skonieczna-Żydecka, Paulina Niedźwiedzka-Rystwej

**Affiliations:** Pomeranian Medical University in Szczecin, Szczecin, Zachodniopomorskie, Poland; Pomeranian Medical University in Szczecin, Szczecin, Zachodniopomorskie, Poland; Pomeranian Medical University in Szczecin, Szczecin, Zachodniopomorskie, Poland; University of Szczecin, Szczecin, Zachodniopomorskie, Poland

## Abstract

**Background:**

Human Immunodeficiency Virus (HIV) infection leads to progressive immune dysfunction, among others mediated through the PD-1/PD-L1 pathway. This study aims to assess the impact of ARV therapy on PD-1 expression and SCORE 2 changes in patients over a twelve-month observation period.

**Figure:**
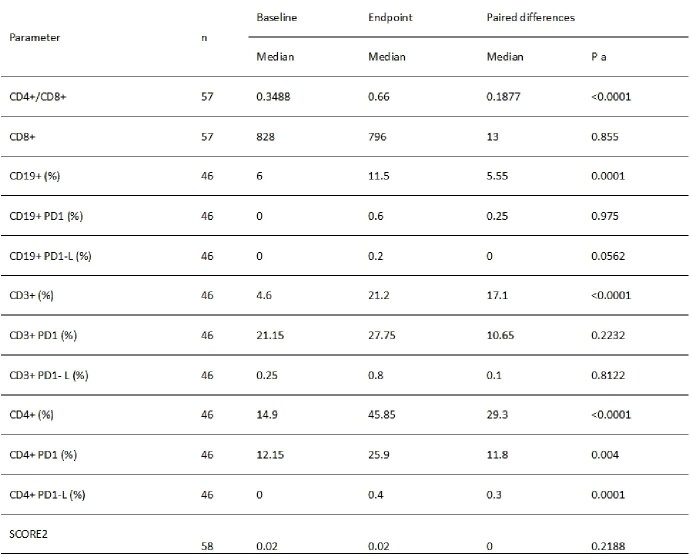

Immunofenotype trends in analysed population.

**Methods:**

This observational study evaluated 58 PLWH undergoing standardized ARV therapy according to national guidelines, obtaining viral suppression. Initial and twelve-month follow-up assessments included measurements of SCORE 2, as well as PD-1 expression, and lymphocyte subsets (CD4+, CD8+, and CD4+/CD8+ ratio) using flow cytometry. Wilcoxon signed-rank tests analyzed within-subject changes in these parameters. Principal Component Analysis (PCA) was employed to reduce dimensionality and identify the major axes of variance in the data (delta for all measures) with ARV type as grouping variable. To determine whether the clusters observed in the PCA scatter plot reflect statistically significant differences among the treatment groups, an Analysis of Variance (ANOVA) was conducted on the scores of the first two principal components.

Figure 1

**Figure:**
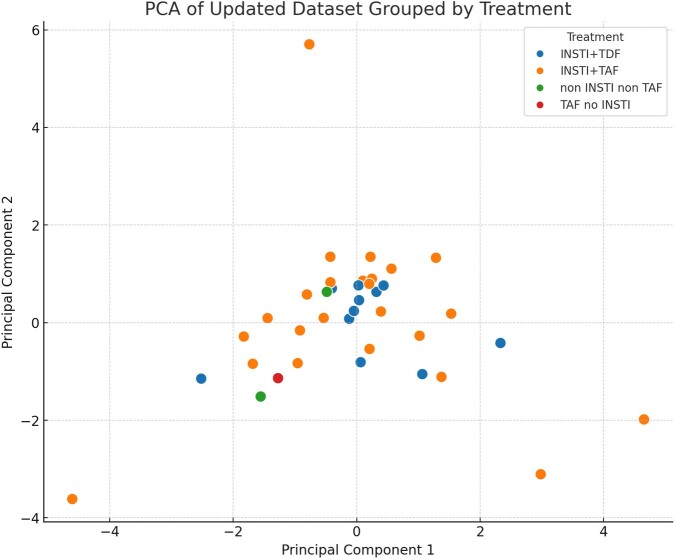

The scatter plot of the first two principal components

**Results:**

There were statistically significant alterations in percentages of CD4+/CD8+ ratio, CD3+, CD4+ along with CD4+ PD-1 and PD-L1 found, all elevated at endpoint. CD19+ was significantly elevated after twelve-month period, as well. Data is displayed in Table 1. We found that the change in these parameters was not dependent on ARV type. SCORE 2 at endpoint correlated positively with CD4+PD1 change (r=0.372; p=0.011), but the parameter change was not dependent on ARV therapy (p >0.05). In PCA analysis, the first principal component captured 22.6% of the total variance, while the second accounted for 20.7%. The scatter plot (figure 1) of the first two principal components showed that the data points (deltas) are distributed and clustered by treatment groups however the differences are not significantly different (PC1: p=0.684; PC2: p=0.840).

**Conclusion:**

Higher expression of PD-1 in CD4+ cells correlates positively with higher cardiovascular risk calculated with SCORE 2, reason for elevated PD1 and PD1-L in ARV treated individuals in comparison to baseline evaluations requires further analysis.

**Disclosures:**

**Bogusz J. Aksak-Wąs, MD. PhD. ID Specialist**, Gilead Sciences: paid lectures|GSK: paid lectures **Miłosz Parczewski, Prof. MD. Phd.**, Gilead Sciences: Advisor/Consultant|Janssen: Advisor/Consultant|MSD: Advisor/Consultant|ViiV: Advisor/Consultant **Karolina Skonieczna-Żydecka, Prof.**, Sanprobi sp. z o.o. sp. k.: Advisor/Consultant|Sanprobi sp. z o.o. sp. k.: Grant/Research Support|Sanprobi sp. z o.o. sp. k.: Honoraria

